# ASO Author Reflections: Omentoplasty to Improve Perineal Wound Healing After Abdominoperineal Resection

**DOI:** 10.1245/s10434-018-6799-5

**Published:** 2018-10-16

**Authors:** Robin D. Blok, Pieter J. Tanis

**Affiliations:** 1 Department of Surgery, Amsterdam UMC, University of Amsterdam, Amsterdam, The Netherlands; 2 LEXOR, Centre for Experimental and Molecular Medicine, Oncode Institute, Cancer Centre Amsterdam, Amsterdam UMC, University of Amsterdam, Amsterdam, The Netherlands

## Past

Abdominoperineal resection (APR) for rectal cancer often is complicated by perineal wound problems.^1^ Some surgeons advocate an omentoplasty to obliterate the pelvic dead space and prevent the small bowel from descending into the pelvis.^2^,^3^ Despite the fact that globally many surgeons routinely construct omentoplasty as part of the APR, only a few small studies support the putative clinical benefits.^4^ This study aimed to evaluate whether omentoplasty reduces early and late perineal complications for patients treated by APR for rectal cancer in a nationwide setting.

## Present

At the population level, no differences in perineal wound healing were observed between patients submitted to omentoplasty and those who were not.^5^ Particularly, patients experienced similar rates of pre-sacral abscess formation and a similar need for reoperation to remove small bowel obstruction in the pelvic cavity. To the contrary, omentoplasty was found to be associated with perineal herniation. This may be explained by the fact that a bulky omentum with a long vascular pedicle exerts more pressure on the perineal scar than a few loops of small bowel with restricted mesenteric length. If confirmed, these findings suggest that perhaps application of omentoplasty for primary filling of the pelvic dead space after APR should be omitted.

## Future

A potential explanation for the inefficacy of omentoplasty in its current nonstandardized and non-quality-controlled application may be related to insufficient obliteration of the pelvic cavity due to inadequate mobilization of the omentum or an insufficient amount of omental fat available. Another reason might be partial ischemia of the omentum after mobilization, leading to partial necrosis and abscess formation of the omentum itself. Therefore, prospective cohort studies should focus on confirming the adequacy of pelvic filling (e.g., by postoperative imaging) and perfusion (e.g., by fluorescence angiography), with subsequent correlation with perineal wound healing. In addition, the technical way to achieve an omentoplasty with optimal filling and perfusion (e.g., left or right gastroepiploic pedicle, tunneling through transverse mesocolon or along the paracolic gutter) also must be determined. If a specific technique of omentoplasty is suggested to have an impact on perineal wound healing, this might subsequently be tested using a randomized study design. Until then, APR without omentoplasty can be considered the standard of care.
